# Public knowledge, attitude and practice on influenxa pandemic (H1N1) 2009 prevention in Southern Vietnam

**DOI:** 10.1186/1753-6561-5-S1-P8

**Published:** 2011-01-10

**Authors:** Ho Thi Thien Ngan, Tran Ngoc Huu, Bui Thu Huong, Nguyen Trung Nghia, Le Van Tuan

**Affiliations:** 1 Pasteur Institute of Ho Chi Minh City, Vietnam; 2 Unilever, Hanoi, Vietnam; 3 Preventive Medicine Center, Can Tho City, Vietnam; 4 World Health Organization, Ho Chi Minh City, Vietnam

## Background

After quickly spreading since March 2009 in Mexico, influenza pandemic H1N1 has affected a large part of the world's population. Countries have made great efforts to contain the pandemic. An important key in containing community transmission and reducing the impacts of the pandemic influenza is to have local people educated to have good knowledge, attitude and practice toward influenza pandemic (H1N1).  The aim of this study was to assess knowledge, attitute and practice of local people toward influenza pandemic (H1N1) prevention after launching education programs since early pandemic period (June 2009).

## Methods

During the post-peak period of influenza pandemic (H1N1) (20 March – 8 April 2010) a cross – sectional survey was conducted in Cu Chi district of Ho Chi Minh City and Ninh Kieu district of Can Tho City. Among 304 individuals (32% male and 68% female) who were selected systematic randomly and representing for risk population as workers, students who living in the boarding houses, boarding schools. Outcome measures were perceived containment and prevention activities, received pandemic information. Knowledge, attitude and practice on personal and community prevention focused on hand washing, respiratory etiquette, avoidance of close contact with sick people, staying home if sick and house cleaning.

## Results

83.9% of respondents felt that the pandemic responses by the government were essential and timely. 87.5% have received the pandemic information through flyers, radio, TV spots, newspaper, and meetings. However, 63.3% felt satisfied with the provided information. Respondents rated face-mask and hand-washing as the most effective preventive measures (97%). The percentage of people who have good knowledge on pandemic influenza personal prevention (i.e. wash hands plus avoid close contact with sick people) was 74.7%, while only 36.8% had good knowledge on community prevention (cover nose and mouth when coughing or sneezing, stay home from work or school if sick, cleaning house). Female had better knowledge on house-cleaning than male, however male had better practice than female with difference statistically significant.

## Conclusion

Implementing education programs were successful and effective in raising public awareness about influenca pandemic. This proven that education measurement is one of important keys of pandemic containment strategies. Although the percentage of people having good knowledge and acceptable attitude were high, practice on community prevention among local people was still poor. Therefore, in the future education programs should focus on improving good practice for containing community transmission of influenza pandemic.

